# Description of 15 novel species within genus Janthinobacterium isolated from glaciers

**DOI:** 10.1099/ijsem.0.007020

**Published:** 2026-01-14

**Authors:** Lei-Lei Yang, Yu-Hua Xin, Qing Liu

**Affiliations:** 1 State Key Laboratory of Microbial Diversity and Innovative Utilization, Institute of Microbiology, Chinese Academy of Sciences, Beijing 100101, PR China; 2 China General Microbiological Culture Collection Center (CGMCC), Institute of Microbiology, Chinese Academy of Sciences, Beijing 100101, PR China; 3 Beijing Key Laboratory of Genetic Element Biosourcing & Intelligent Design for Biomanufacturing, Beijing 100101, PR China

**Keywords:** glacier, *Janthinobacterium*, psychrotolerant

## Abstract

Fifteen Gram-stain-negative, rod-shaped bacterial strains, motile by means of a monopolar flagellum, were isolated from glaciers located on the Tibetan Plateau, P.R. China. These strains, designated MDT1-19^T^, MDB2-8^T^, HLS12-2^T^, HLX7-2^T^, LB2P49^T^, LB2P70^T^, LB3P112^T^, LB3P118^T^, LB2P10^T^, RB2R34^T^, ZB1P44^T^, RB2P8^T^, GB1R12^T^, GB4P2^T^ and RT4P48^T^, belong to the genus *Janthinobacterium* based on 16S rRNA gene sequences analysis. The average nucleotide identity and digital DNA–DNA hybridization values between these strains and species with validly published names were below 96% and 70%, respectively. Based on the phylogenetic, genotypic and phenotypic evidence, these 15 strains are proposed to represent fifteen novel species within the genus *Janthinobacterium*, with the following proposed names: *Janthinobacterium algoris* sp. nov. (MDT1-19^T^=CGMCC 1.9797=NBRC 116353^T^), *Janthinobacterium breve* sp. nov. (MDB2-8^T^=CGMCC 1.9853^T^=NBRC 116354^T^), *Janthinobacterium darwini* sp. nov. (HLS12-2^T^=CGMCC 1.9980^T^=NBRC 116355^T^), *Janthinobacterium woesei* sp. nov. (HLX7-2^T^=CGMCC 1.9990^T^=NBRC 116356^T^), *Janthinobacterium salmonicoloratum* sp. nov. (LB2P49^T^=CGMCC 1.11246^T^=NBRC 116357^T^), *Janthinobacterium violaceum* sp. nov. (LB2P70^T^=CGMCC 1.11252^T^=NBRC 116358^T^), *Janthinobacterium leeuwenhoeki* sp. nov. (LB3P112^T^=CGMCC 1.11294^T^=NBRC 116359^T^), *Janthinobacterium pasteuri* sp. nov. (LB3P118^T^=CGMCC 1.11299^T^=NBRC 116360^T^), *Janthinobacterium paucivorans* sp. nov. (LB2P10^T^=CGMCC 1.11332^T^=NBRC 116361^T^), *Janthinobacterium longum* sp. nov. (RB2R34^T^=CGMCC 1.11894^T^=NBRC 116362^T^), *Janthinobacterium glycogeni* sp. nov. (ZB1P44^T^=CGMCC 1.23234^T^=NBRC 116363^T^), *Janthinobacterium kochi* sp. nov. (RB2P8^T^=CGMCC 1.23776^T^=NBRC 116364^T^), *Janthinobacterium goodfellowi* sp. nov. (GB1R12^T^=CGMCC 1.24272^T^=NBRC 116365^T^), *Janthinobacterium lyxosi* sp. nov. (GB4P2^T^=CGMCC 1.24307^T^=NBRC 116366^T^) and *Janthinobacterium amylolyticum* sp. nov. (RT4P48^T^=CGMCC 1.24355^T^=NBRC 116367^T^).

The genus *Janthinobacterium*, established by De Ley *et al.* in 1978 [1] and later refined by Lincoln *et al.* in 1999 [2], encompasses Gram-negative, rod-shaped, aerobic and motile bacteria within the phylum *Pseudomonadota*. As of 3 August 2025, only eight species have validly published names, with three additional proposed species awaiting validation [3]. Most species were isolated from aquatic environments, such as *Janthinobacterium aestuarii* [4], *Janthinobacterium aquaticum* [5], *Janthinobacterium violaceinigrum* [5], *Janthinobacterium rivuli* [5], *Janthinobacterium fluminis* [6], and *Janthinobacterium kumbetense* [7]. *Janthinobacterium* strains contain ubiquinone Q-8 as the primary respiratory quinone and a fatty acid profile featuring C_16:0_, C_16:1_* *ω*7c* and C_18:1_* *ω*7c*. Certain species produce violacein, a purple pigment with antimicrobial properties, highlighting their ecological and biotechnological significance [7]. The limited number of recognized *Janthinobacterium* species highlights the potential for discovering novel taxa, particularly from extreme habitats, such as high-altitude glaciers, which remain underexplored microbial reservoirs. This study reports the isolation of 15 novel *Janthinobacterium* species from glacier surfaces in China. It aims to characterize these species through a polyphasic taxonomic approach, integrating phenotypic, genomic and phylogenomic analyses. This discovery expands the known diversity of *Janthinobacterium* and emphasizes the ecological importance of glacial ecosystems.

## Isolation and ecology

Samples were collected in October 2009 from the Midui Glacier (Xizang), in January 2011 from Hailuogou Glacier (Sichuan) and in October 2016 from the Laigu, Renlongba, Zepu and Gawalong glaciers (Xizang). To minimize contamination, aseptic protocols were strictly followed during sample collection, including the use of sterile gloves. Subsurface ice and cryoconite samples were precisely extracted with a sterilized spade and were expedited to the facility via a cryogenic transport unit containing dry ice. Serial dilutions in autoclaved H₂O were performed, followed by plating on Reasoner’s 2A medium (BD Difco) and peptone, yeast extract and glucose (PYG) medium [0.5% (w/v) Bacto peptone (Difco), 0.02% (w/v) yeast extract, 0.5% (w/v) glucose, 0.3% (w/v) beef extract, 0.05% (w/v) NaCl, 0.15% (w/v) MgSO_4_·7H_2_O, pH 7.0]. After incubation at 14 °C, more than 2,000 bacterial isolates were collected. In this study, five strains (MDT1-19^T^, MDB2-8^T^, HLS12-2^T^, HLX7-2^T^ and RT4P48^T^) were isolated from cryoconite samples. The remaining ten strains (LB2P49^T^, LB2P70^T^, LB3P112^T^, LB3P118^T^, LB2P10^T^, RB2R34^T^, ZB1P44^T^, RB2P8^T^, GB1R12^T^ and GB4P2^T^) were derived from ice samples (Table S1, available in the online Supplementary Material). These strains were preserved in 10% (v/v) glycerol suspensions in liquid nitrogen.

## 16S rRNA phylogeny

Genomic DNA was isolated using the TaKaRa MiniBEST Bacteria Genomic DNA Extraction Kit ver. 3.0 (TaKaRa, Japan) following the manufacturer’s instructions. The 16S rRNA gene was amplified and sequenced with the universal primers 27F and 1492R [8]. Sequence analysis was conducted using the EzBioCloud database [9]. Multiple alignments were generated with the clustal_w program implemented in mega v.12 [10], and phylogenetic trees were constructed using the neighbour-joining (NJ) [11] method in mega. Genetic distances for the NJ analysis were computed with Kimura’s two-parameter model [12], and tree robustness was assessed with 1,000 bootstrap replicates [13].

The 16S rRNA gene sequences of these 15 strains were obtained from PCR products and compared to 16S rRNA gene sequences from other bacterial species using the EzBioCloud server, revealing that they belong to the genus *Janthinobacterium*. High 16S rRNA gene sequence similarities (98.9–100%) were observed among the 15 strains (Table S2). They showed 99.2–99.93% similarity to ‘*Janthinobacterium tructae*’ SNU WT3^T^, ‘*Janthinobacterium svalbardensis*’ JA-1^T^, *Janthinobacterium lividum* DSM 1522^T^, *J. rivuli* FT68W^T^, ‘*Janthinobacterium psychrotolerans*’ S3-2^T^ and ‘*J. kumbetense*’ GK^T^ (Table S3). A phylogenetic tree constructed using the NJ method showed that strains LB2P49^T^ and RB2P8^T^ formed a small clade, LB2P70^T^ and RT4P48^T^ clustered with *J. kumbetense* GK^T^, ZB1P44^T^ grouped with ‘*J. psychrotolerans*’ S3-2^T^, while strains HLS12-2^T^, LB3P112^T^, LB3P118^T^ and LB2P10^T^ clustered together. The remaining six strains formed a distinct branch, separated from other relatives (Fig. S1). However, most nodes of the phylogenetic tree exhibited low bootstrap support, indicating limitations in phylogenetic analysis of the genus *Janthinobacterium* based on the 16S rRNA gene sequence.

## Genome features

Genome sequencing of the 15 strains was performed on the Illumina HiSeq 4000 platform (Illumina, San Diego, CA, USA), producing 150 bp paired-end reads following the manufacturer’s instructions. Short reads were assembled *de novo* into draft genomes using SPAdes [14]. Genome quality was assessed for completeness and contamination using CheckM2 v.1.0.2 [15] and QUAST v.5.2 [16]. Annotation was carried out with Bakta version 1.7.0 [17]. Average nucleotide identity (ANI) values were determined with FastANI [18], and digital DNA–DNA hybridization (dDDH) values were calculated via the Type (Strain) Genome Server [19]. Genomic sequences representing *Janthinobacterium* species were selected from the GTDB Release R220 database [20] for ANI calculation, alongside species with validly published names. Clustering of the pairwise ANI values was performed using the ‘bactaxR’ package in R with the average linkage hierarchical clustering method [21]. A set of 81 core genes was retrieved using the UBCG2 program [22], aligned with MAFFT v.7.520 [23], and used to generate a maximum likelihood (ML) [24] phylogenomic tree in IQ-TREE v.2.3.4 [25]. The tree, constructed from concatenated core gene sequences, utilized the GTR+F+I+R5 substitution model and was supported by 1,000 bootstrap replicates. Predicted protein sequences were assigned to Clusters of Orthologous Groups (COG) categories using blast+ [26] against the latest COG database [27].

Genomic sequencing yielded high-quality assemblies for all 15 strains (Table S4). Genome completeness reached 100%, with contamination levels ranging from 0 to 1.4%. The full-length 16S rRNA gene extracted from each genome matched its corresponding PCR product exactly. Their genome sizes ranged from 4.90 Mb (LB2P10^T^) to 6.44 Mb (MDB2-8^T^), with contig counts between 15 and 88 and G+C content between 60.8% and 63.3%. Genome annotation details are listed in Table S5. Each strain contained 4,225–5,664 CDSs, 10–15 ncRNAs, 75–85 tRNAs, 4–9 rRNAs and one tmRNA. In terms of COG functional classification, these strains processed an average of 817 genes for information storage and processing, 1,458 genes for cellular processes and signalling and 1,792 genes for metabolism, showing a pattern similar to that of the type strains of the genus *Janthinobacterium*.

**Fig. 1.**
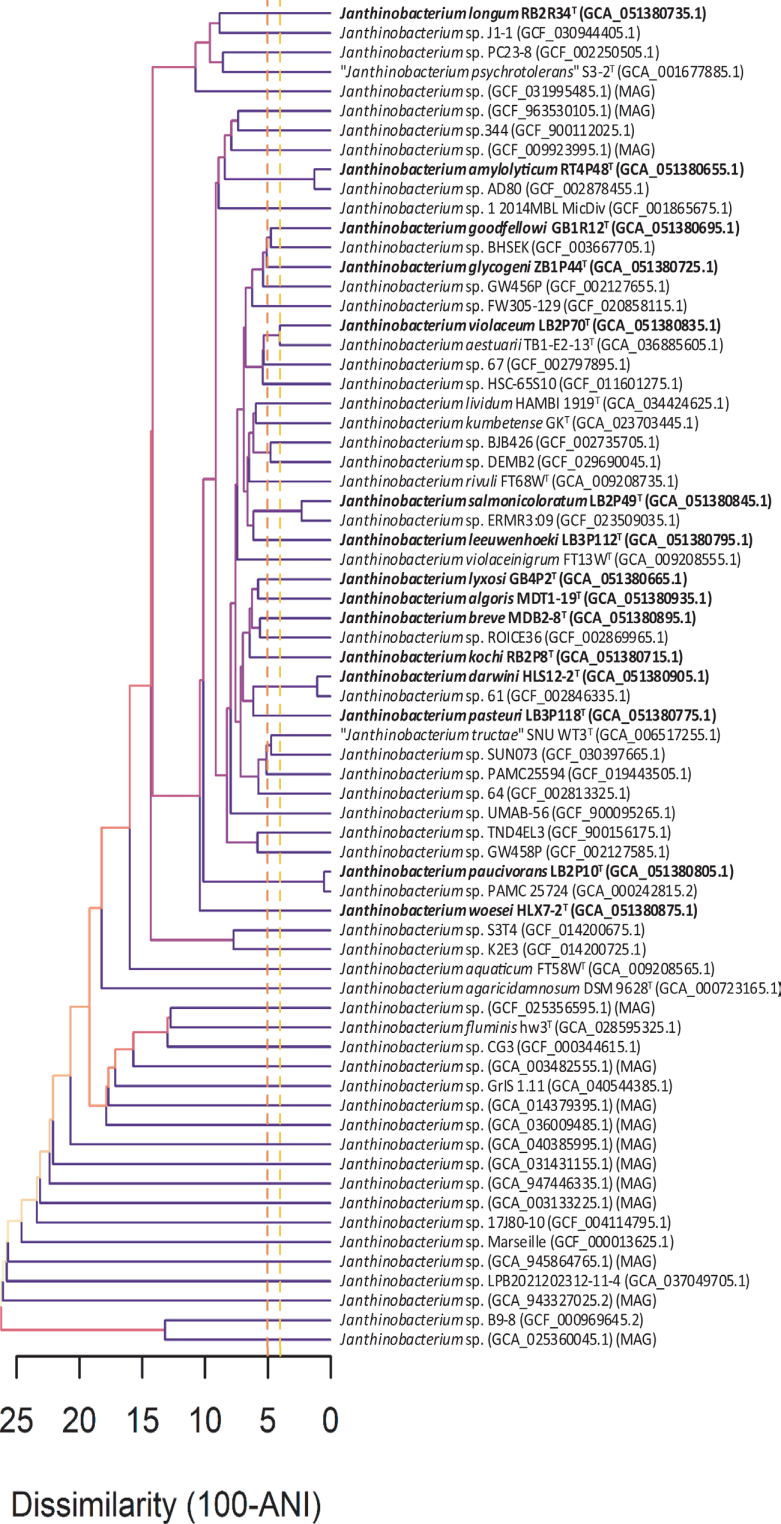
Cluster analysis based on pairwise ANI values of the 15 strains and related strains in the genus *Janthinobacterium*. Accession numbers of the genomic sequences are provided in parentheses.

A total of 54 genomic sequences representing 54 *Janthinobacterium* species, including 40 isolates and 14 metagenome-assembled genomes (MAGs), were retrieved from the GTDB Release R220 database and the National Center for Biotechnology Information (NCBI) database. Pairwise ANI values of the genomes of 15 new isolates and 54 related genomic sequences were calculated and subjected to clustered analysis. The clustering results, shown in Fig. 1, indicated that the 69 genomic sequences represented 64 species, far exceeding the number of published species in the genus *Janthinobacterium*, highlighting the underestimated diversity of *Janthinobacterium* in the environment. Strains RT4P48^T^, LB2P49^T^, HLS12-2^T^ and LB2P10^T^ clustered individually with *Janthinobacterium* sp. AD80, *Janthinobacterium* sp. ERMR3:09, *Janthinobacterium* sp. 61 and *Janthinobacterium* sp. PAMC 25724, respectively, with ANI values ≥96%, indicating that each corresponding pair represents the same species [28]. Strains *Janthinobacterium* sp. ERMR3:09 and *Janthinobacterium* sp. PAMC 25724 were also collected from glacier samples from India and the Alps mountains, respectively, suggesting that this study identified the same species in similar glacial environments on the Tibetan Plateau. No genomic sequences exceeded a 96% ANI value with the other 11 strains, confirming their novelty within the genus *Janthinobacterium* in both cultured isolates and MAGs. The pairwise ANI values of the 15 strains were also calculated and listed in Table S2, showing that all values were below 95%, confirming they represented different species.

To further confirm whether these strains belong to novel species, pairwise dDDH values were calculated and a genome blast distance phylogeny was conducted between them and 10 published species (Fig. S2). Pairwise dDDH values between the 15 strains ranged from 26.2% to 55.0%, and those between the strains and related type strains ranged from 22.2% to 64.0%, with the highest value observed between strain LB2P70^T^ and *J. aestuarii* TB1-E2-13^T^. These values were below bacterial species threshold (dDDH <70%) [29], confirming that they represent 15 novel species of genus *Janthinobacterium*.

**Fig. 2.**
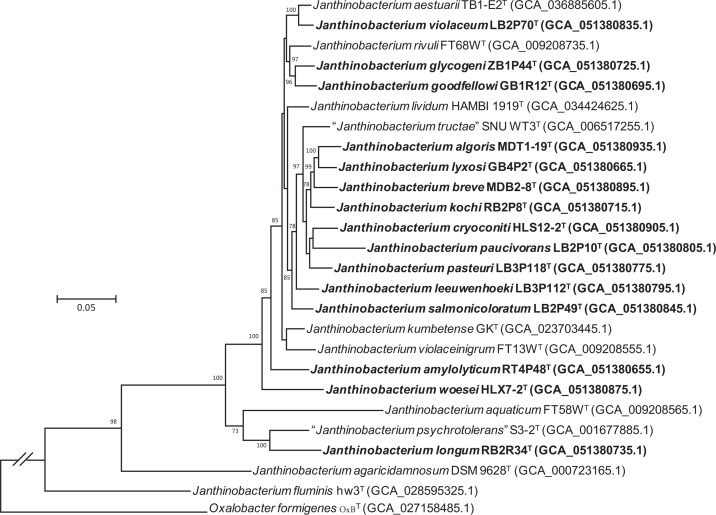
Phylogenetic tree of the 15 strains and related taxa inferred using ML algorithms in IQ-TREE based on the concatenated alignment of 81 core genes. Bootstrap values (>50%) based on 1,000 replicates are shown at the branch nodes. Scale bar, 0.05 substitutions per nucleotide position.

Unlike the 16S rRNA phylogenetic tree, which was difficult to distinguish multiple strains and had low bootstrap support, the phylogenomic tree clearly distinguished the 15 strains from related taxa, each with a separate branch (Fig. 2). Strains LB2P70^T^ and *J. aestuarii* TB1-E2-13^T^ formed a small cluster. Strains ZB1P44^T^ and GB1R12^T^ grouped with 96% bootstrap value and are most related to *J. rivuli* FT68W^T^. Strains MDT1-19^T^, GB4P2^T^, MDB2-8^T^, RB2P8^T^, HLS12-2^T^, LB2P10^T^ and LB3P118^T^ formed a clade and clustered with ‘*J. tructae*’ SNU WT3^T^ with 97% bootstrap value. Strain RB2R34^T^ clustered with ‘*J. psychrotolerans*’ S3-2^T^ and *J. aquaticum* FT58WT. Strains LB3P112^T^, LB2P49^T^, RT4P48^T^ and HLX7-2^T^ formed independent phylogenetic clades, separate from other strains, supporting the classification of these strains as novel species within the genus *Janthinobacterium*.

## Physiology

Colony morphology was examined on PYG plates, Gram-staining followed established methods [30], and cell structure was analysed using JEM-1400 transmission electron microscopy (JEOL Ltd., Tokyo, Japan). Growth was tested across 4–37 °C in PYG medium, with pH tolerance (4.0–11.0, 1.0-unit increments) evaluated in PYG broth adjusted with 0.2 M Na_2_HPO_4_/NaH_2_PO_4_ buffer (pH 5–8) or Na_2_CO_3_/NaHCO_3_ buffer (pH 9–10). NaCl tolerance [0–6% (w/v), in 0.5% increments] was assessed in PYG broth. Hydrolysis of starch, casein, tributyrin and Tween 80 was tested on PYG plates per Smibert and Krieg’s protocol [31]. Catalase activity was detected by bubble formation in 3% (v/v) H_2_O_2_, and oxidase activity was confirmed with 1% (w/v) tetramethyl-p-phenylenediamine. Carbon utilization was tested using API 50CH strips (bioMérieux) (Marcy-l’Étoile, France) with a basal medium [0.2% (w/v) (NH_4_)_2_SO_4_, 0.05% (w/v) NaH_2_PO4·H_2_O, 0.05% (w/v) K_2_HPO4, 0.02% (w/v) MgSO_4_·7H_2_O and 0.01% (w/v) CaCl_2_·2H_2_O]. Acid production was assessed using the API 50CH strip with API 50 CHB medium. Enzyme activities and biochemical properties were evaluated using API 20NE, API 20E and API ZYM strips according to the manufacturer’s instructions.

All 15 isolates were psychrotolerant, growing at 4 °C. Colonies displayed diverse colours, appearing white, pale yellow, salmon or violet. Each strain possessed a single polar flagellum and exhibited motility (Fig. S3). All isolates tested positive for nitrate reduction to nitrite, citrate utilization and the Voges–Proskauer reaction. They consistently expressed arginine dihydrolase, alkaline phosphatase, leucine arylamidase, valine arylamidase, acid phosphatase, naphthol-AS-BI-phosphohydrolase and *α*-glucosidase. All strains could utilize glycerol, d-ribose, d-glucose, d-fructose, d-mannose, myo-inositol and d-maltose as a sole carbon source. Starch hydrolysis was negative for all strains except RT4P48^T^. Beyond these shared characteristics, significant inter-strain heterogeneity was observed in gelatin hydrolysis, aesculin hydrolysis, glucose fermentation, enzymatic profiles and carbon source utilization (Table 1). These phenotypic variations highlight the evolution of divergent metabolic and physiological traits within a single ecological niche, which can be used to differentiate species. Strain LB2P70ᵀ can also be distinguished from its closest relative, *J. aestuarii* TB1-E2-13^T^: strain LB2P70ᵀ grows over a broader pH (5.0–10.0) and NaCl concentration range (0–2.5%, w/v); is positive for casein hydrolysis, the Voges–Proskauer test and urease; and utilizes d-fructose, whereas *J. aestuarii* TB1-E2-13^T^ lacks these activities (Table S6).

**Table 1.** Differential characteristic phenotype of the 15 *Janthinobacterium* strains isolated in this study Strains: 1, MDT1-19^T^; 2, MDB2-8^T^; 3, HLS12-2^T^; 4, HLX7-2^T^; 5, LB2P49^T^; 6, LB2P70^T^; 7, LB3P112^T^; 8, LB3P118^T^; 9, LB2P10^T^; 10, RB2R34^T^; 11, ZB1P44^T^; 12, RB2P8^T^; 13, GB1R12^T^; 14, GB4P2^T^; 15, RT4P48^T^. +, Positive; –, negative.

Characteristic	1	2	3	4	5	6	7	8	9	10	11	12	13	14	15
Starch hydrolysis	−	−	−	−	−	−	−	−	−	−	−	−	−	−	+
Casein hydrolysis	+	+	−	−	+	+	−	−	−	−	+	+	+	−	−
Gelatin hydrolysis	+	+	−	+	+	+	+	+	+	−	+	+	+	+	+
Aesculin hydrolysis	+	+	+	+	+	+	+	+	−	+	+	−	+	+	+
Glucose fermentation	−	−	−	−	+	−	+	+	−	+	−	−	−	+	−
**Enzyme activity**															
Urease	+	−	+	+	+	+	+	+	+	+	+	+	+	−	+
*β*-Galactosidase	−	−	−	−	−	−	−	−	−	+	−	−	−	−	−
Lysine decarboxylase	+	+	−	−	+	+	−	−	−	+	+	+	+	−	+
Esterase (C4)	−	+	−	+	+	+	+	+	+	+	+	+	+	+	+
Esterase lipase (C8)	−	−	−	−	+	+	+	−	+	+	+	−	+	−	−
Cystine arylamidase	−	−	+	−	−	−	+	−	−	+	+	−	−	−	−
*α*-Chymotrypsin	+	+	−	+	+	+	+	−	−	−	−	−	−	+	+
**Utilization of**															
d-Arabinose	−	+	+	+	+	+	−	−	−	+	+	+	+	+	−
d-Xylose	+	+	+	+	+	+	−	−	−	+	+	+	+	+	+
l-Xylose	+	−	+	+	+	+	−	−	−	−	−	−	+	−	−
d-Adonitol	−	−	−	+	−	−	−	−	−	−	−	−	−	−	+
l-Rhamnose	−	−	−	+	−	+	−	−	−	+	−	−	−	+	−
d-Sorbitol	+	+	+	+	+	+	+	−	−	−	+	+	+	−	+
Arbutin	+	+	+	+	+	+	−	+	−	+	+	−	+	+	+
Salicin	+	+	+	+	+	+	−	+	−	+	+	−	+	+	+
d-Cellobiose	+	+	+	+	+	+	−	+	−	+	+	+	+	+	+
d-Lactose	+	+	+	−	+	+	−	−	−	+	+	+	+	−	+
d-Sucrose	+	+	+	−	+	+	+	−	−	+	+	+	+	−	−
Inulin	−	−	−	−	−	+	−	−	−	+	−	−	−	−	−
d-Raffinose	−	−	−	−	−	+	−	−	−	+	−	−	−	−	−
Glycogen	−	−	−	−	−	−	−	−	−	−	+	−	−	−	−
Xylitol	+	+	+	+	+	+	+	−	−	−	+	+	+	−	+
d-Fucose	−	−	−	−	−	−	−	−	−	−	−	−	−	+	−
l-Fucose	−	+	+	+	+	+	−	−	−	+	+	+	+	+	−
d-Arabitol	+	+	+	+	+	+	+	−	−	+	+	+	+	−	+
l-Arabitol	−	−	−	+	−	−	−	−	−	−	−	−	−	−	+
Gluconate	−	+	−	−	+	−	+	+	−	−	+	−	+	+	−

Based on phylogenetic, physiological and genotypic features, strains MDT1-19^T^, MDB2-8^T^, HLS12-2^T^, HLX7-2^T^, LB2P49^T^, LB2P70^T^, LB3P112^T^, LB3P118^T^, LB2P10^T^, RB2R34^T^, ZB1P44^T^, RB2P8^T^, GB1R12^T^, GB4P2^T^ and RT4P48^T^ were classified as fifteen novel species of the genus *Janthinobacterium*, with the following proposed names: *Janthinobacterium algoris* sp. nov. (MDT1-19^T^=CGMCC 1.9797=NBRC 116353^T^), *Janthinobacterium breve* sp. nov. (MDB2-8^T^=CGMCC 1.9853^T^=NBRC 116354^T^), *Janthinobacterium darwini* sp. nov. (HLS12-2^T^=CGMCC 1.9980^T^=NBRC 116355^T^), *Janthinobacterium woesei* sp. nov. (HLX7-2^T^=CGMCC 1.9990^T^=NBRC 116356^T^), *Janthinobacterium salmonicoloratum* sp. nov. (LB2P49^T^=CGMCC 1.11246^T^=NBRC 116357^T^), *Janthinobacterium violaceum* sp. nov. (LB2P70^T^=CGMCC 1.11252^T^=NBRC 116358^T^), *Janthinobacterium leeuwenhoeki* sp. nov. (LB3P112^T^=CGMCC 1.11294^T^=NBRC 116359^T^), *Janthinobacterium pasteuri* sp. nov. (LB3P118^T^=CGMCC 1.11299^T^=NBRC 116360^T^), *Janthinobacterium paucivorans* sp. nov. (LB2P10^T^=CGMCC 1.11332^T^=NBRC 116361^T^), *Janthinobacterium longum* sp. nov. (RB2R34^T^=CGMCC 1.11894^T^=NBRC 116362^T^), *Janthinobacterium glycogeni* sp. nov. (ZB1P44^T^=CGMCC 1.23234^T^=NBRC 116363^T^), *Janthinobacterium kochi* sp. nov. (RB2P8^T^=CGMCC 1.23776^T^=NBRC 116364^T^), *Janthinobacterium goodfellowi* sp. nov. (GB1R12^T^=CGMCC 1.24272^T^=NBRC 116365^T^), *Janthinobacterium lyxosi* sp. nov. (GB4P2^T^=CGMCC 1.24307^T^=NBRC 116366^T^) and *Janthinobacterium amylolyticum* sp. nov. (RT4P48^T^=CGMCC 1.24355^T^=NBRC 116367^T^).

## Description of *Janthinobacterium algoris* sp. nov.

*Janthinobacterium algoris* (al’go.ris. L. gen. n. *algoris*, of the cold)

Cells are Gram-stain-negative, aerobic and motile by means of a monopolar flagellum, measuring 1.5–2.0 µm in length and 0.9–1.0 µm in width. Colonies are white-coloured, round and slightly convex. Growth occurs at 4–33 °C (optimum, 25–28 °C), pH 4.0–10.0 (optimum, pH 7.0) and 0–2.0% (w/v) NaCl. Positive for oxidase and catalase. Hydrolyse casein, gelatin and aesculin, but not starch and Tween 80. Positive for reduction of nitrate to nitrite, citrate utilization, Voges–Proskauer test, urease, arginine dihydrolase, lysine decarboxylase, alkaline phosphatase, leucine arylamidase, valine arylamidase, *α*-chymotrypsin, acid phosphatase, naphthol-AS-BI-phosphohydrolase, *α*-glucosidase and *β*-glucosidase. Negative for indole production, H_2_S production, fermentation of glucose, *β*-galactosidase, ornithine decarboxylase, tryptophan deaminase, esterase (C4), esterase lipase (C8), lipase (C14), cystine arylamidase, trypsin, *α*-galactosidase, *β*-glucuronidase, *N*-acetyl-*β*-glucosaminidase, *α*-mannosidase and *α*-fucosidase. Utilizes the following carbohydrate as the sole carbon source: glycerol, l-arabinose, d-ribose, d-xylose, l-xylose, d-galactose, d-glucose, d-fructose, d-mannose, inositol, d-mannitol, d-sorbitol, *N*-acetylglucosamine, arbutin, aesculin, salicin, d-cellobiose, d-maltose, d-lactose, d-sucrose, xylitol, d-lyxose, d-arabitol and 2-ketogluconate. Cannot utilize the following carbohydrates: erythritol, d-arabinose, d-adonitol, methyl-*β*-d-xylopyranoside, l-sorbose, l-rhamnose, dulcitol, methyl-*α*-d-mannopyranoside, methyl-*α*-d-glucopyranoside, amygdaline, d-melibiose, d-trehalose, inulin, d-melezitose, d-raffinose, starch, glycogen, gentiobiose, d-turanose, d-tagatose, d-fucose, l-fucose, l-arabitol, gluconate and 5-ketogluconate. Acids are produced from d-galactose, d-mannose, aesculin and d-cellobiose. The assembled genome of the type strain is 6.40 Mb in size, with a G+C content of 61.6%.

The type strain MDT1-19^T^ (= CGMCC 1.9797^T^=NBRC 116353^T^) was isolated from a cryoconite sample collected from the Midui glacier on the Tibetan Plateau, P.R. China. The NCBI accession numbers for the 16S rRNA gene and genome sequences are JX949578 and JBNYXZ000000000, respectively.

## Description of *Janthinobacterium breve* sp. nov.

*Janthinobacterium breve* (bre’ve. L. neut. adj. *breve*, short, the shape of the cell)

Cells are Gram-stain-negative, aerobic and motile by means of a monopolar flagellum, measuring 1.2–1.8 µm in length and 0.9–1.1 µm in width. Colonies are light yellow-coloured, round and slightly convex. Growth occurs at 4–34 °C (optimum, 25–28 °C), pH 5.0–10.0 (optimum, pH 7.0) and 0–2.0% (w/v) NaCl. Positive for oxidase and catalase. Hydrolyse casein, gelatin and aesculin, but not starch and Tween 80. Positive for reduction of nitrate to nitrite, citrate utilization, Voges–Proskauer test, arginine dihydrolase, lysine decarboxylase, ornithine decarboxylase, alkaline phosphatase, esterase (C4), leucine arylamidase, valine arylamidase, *α*-chymotrypsin, acid phosphatase, naphthol-AS-BI-phosphohydrolase, *α*-glucosidase and *β*-glucosidase. Negative for indole production, H_2_S production, fermentation of glucose, urease, *β*-galactosidase, tryptophan deaminase, esterase lipase (C8), lipase (C14), cystine arylamidase, trypsin, *α*-galactosidase, *β*-glucuronidase, *N*-acetyl-*β*-glucosaminidase, *α*-mannosidase and *α*-fucosidase. Utilizes the following carbohydrate as the sole carbon source: glycerol, d-arabinose, l-arabinose, d-ribose, d-xylose, d-galactose, d-glucose, d-fructose, d-mannose, inositol, d-mannitol, d-sorbitol, *N*-acetylglucosamine, arbutin, aesculin, salicin, d-cellobiose, d-maltose, d-lactose, d-sucrose, starch, xylitol, d-lyxose, l-fucose, d-arabitol, gluconate and 2-ketogluconate. Cannot utilize the following carbohydrates: erythritol, l-xylose, d-adonitol, methyl-*β*-d-xylopyranoside, l-sorbose, l-rhamnose, dulcitol, methyl-*α*-d-mannopyranoside, methyl-*α*-d-glucopyranoside, amygdaline, d-melibiose, d-trehalose, inulin, d-melezitose, d-raffinose, glycogen, gentiobiose, d-turanose, d-tagatose, d-fucose, l-arabitol and 5-ketogluconate. Acids are produced from d-arabinose, l-arabinose, d-glucose, d-fructose, d-mannose, inositol, d-mannitol, d-sorbitol, aesculin, d-lactose and d-sucrose. The assembled genome of the type strain is 6.44 Mb in size, with a G+C content of 61.9%.

The type strain MDB2-8^T^ (= CGMCC 1.9853^T^=NBRC 116354^T^) was isolated from a cryoconite sample collected from the Midui glacier on the Tibetan Plateau, P.R. China. The NCBI accession numbers for the 16S rRNA gene and genome sequences are JX949582 and JBNYXY000000000, respectively.

## Description of *Janthinobacterium darwini* sp. nov.

*Janthinobacterium darwini* (dar.wi’ni. N.L. gen. n. *darwini*, of Charles Robert Darwin)

Cells are Gram-stain-negative, aerobic and motile by means of a monopolar flagellum, measuring 1.7–1.9 µm in length and 0.9–1.0 µm in width. Colonies are white-coloured, round and slightly convex. Growth occurs at 4–33 °C (optimum, 25–28 °C), pH 5.0–10.0 (optimum, pH 7.0) and 0–3.5% (w/v) NaCl. Positive for oxidase and catalase. Hydrolyse aesculin, but not casein, gelatin, starch and Tween 80. Positive for reduction of nitrate to nitrite, citrate utilization, Voges–Proskauer test, urease, arginine dihydrolase, ornithine decarboxylase, alkaline phosphatase, leucine arylamidase, valine arylamidase, acid phosphatase, cystine arylamidase, naphthol-AS-BI-phosphohydrolase, *α*-glucosidase and *β*-glucosidase. Negative for indole production, H_2_S production, fermentation of glucose, *β*-galactosidase, lysine decarboxylase, tryptophan deaminase, esterase (C4), esterase lipase (C8), lipase (C14), trypsin, *α*-chymotrypsin, *α*-galactosidase, *β*-glucuronidase, *N*-acetyl-*β*-glucosaminidase, *α*-mannosidase and *α*-fucosidase. Utilizes the following carbohydrate as the sole carbon source: glycerol, d-arabinose, l-arabinose, d-ribose, d-xylose, l-xylose, d-galactose, d-glucose, d-fructose, d-mannose, inositol, d-mannitol, d-sorbitol, *N*-acetylglucosamine, arbutin, aesculin, salicin, d-cellobiose, d-maltose, d-lactose, d-sucrose, starch, xylitol, d-lyxose, l-fucose, d-arabitol and 2-ketogluconate. Cannot utilize the following carbohydrates: erythritol, d-adonitol, methyl-*β*-d-xylopyranoside, l-sorbose, l-rhamnose, dulcitol, methyl-*α*-d-mannopyranoside, methyl-*α*-d-glucopyranoside, amygdaline, d-melibiose, d-trehalose, inulin, d-melezitose, d-raffinose, glycogen, gentiobiose, d-turanose, d-tagatose, d-fucose, l-arabitol, gluconate and 5-ketogluconate. Acids are produced from glycerol, d-arabinose, l-arabinose, d-ribose, d-xylose, d-galactose, d-glucose, d-fructose, d-mannose, inositol, d-mannitol, d-sorbitol, aesculin, d-maltose, d-sucrose, d-lyxose, l-fucose and d-arabitol. The assembled genome of the type strain is 5.85 Mb in size, with a G+C content of 61.9%.

The type strain HLS12-2^T^ (= CGMCC 1.9980^T^=NBRC 116355^T^) was isolated from a cryoconite sample collected from the Hailuogou glacier in Sichuan province, P.R. China. The NCBI accession numbers for the 16S rRNA gene and genome sequences are JX949441 and JBNYXX000000000, respectively.

## Description of *Janthinobacterium woesei* sp. nov.

*Janthinobacterium woesei* (woe’se.i. N.L. gen. n. *woesei*, of Carl R. Woese)

Cells are Gram-stain-negative, aerobic and motile by means of a monopolar flagellum, measuring 1.4–2.0 µm in length and 0.8–1.0 µm in width. Colonies are white-coloured, round and slightly convex. Growth occurs at 4–28 °C (optimum, 25 °C), pH 5.0–10.0 (optimum, pH 7.0) and 0–2.0% (w/v) NaCl. Positive for oxidase and catalase. Hydrolyse gelatin and aesculin, but not casein, starch and Tween 80. Positive for reduction of nitrate to nitrite, citrate utilization, Voges–Proskauer test, urease, arginine dihydrolase, alkaline phosphatase, esterase (C4), leucine arylamidase, valine arylamidase, *α*-chymotrypsin, acid phosphatase, naphthol-AS-BI-phosphohydrolase, *α*-glucosidase and *β*-glucosidase. Negative for indole production, H_2_S production, fermentation of glucose, *β*-galactosidase, lysine decarboxylase, ornithine decarboxylase, tryptophan deaminase, esterase lipase (C8), lipase (C14), cystine arylamidase, trypsin, *α*-galactosidase, *β*-glucuronidase, *N*-acetyl-*β*-glucosaminidase, *α*-mannosidase and *α*-fucosidase. Utilizes the following carbohydrate as the sole carbon source: glycerol, d-arabinose, l-arabinose, d-ribose, d-xylose, l-xylose, d-adonitol, d-galactose, d-glucose, d-fructose, d-mannose, l-rhamnose, inositol, d-mannitol, d-sorbitol, arbutin, aesculin, salicin, d-cellobiose, d-maltose, xylitol, d-lyxose, l-fucose, d-arabitol, l-arabitol and 2-ketogluconate. Cannot utilize the following carbohydrates: erythritol, methyl-*β*-d-xylopyranoside, l-sorbose, dulcitol, methyl-*α*-d-mannopyranoside, methyl-*α*-d-glucopyranoside, *N*-acetylglucosamine, amygdaline, d-lactose, d-melibiose, d-sucrose, d-trehalose, inulin, d-melezitose, d-raffinose, starch, glycogen, gentiobiose, d-turanose, d-tagatose, d-fucose, gluconate and 5-ketogluconate. Acids are produced from glycerol, d-arabinose, l-arabinose, d-ribose, d-xylose, d-adonitol, d-galactose, d-glucose, d-fructose, d-mannose, l-rhamnose, inositol, d-mannitol, d-sorbitol, aesculin, d-maltose, d-lyxose, l-fucose, d-arabitol and l-arabitol. The assembled genome of the type strain is 5.63 Mb in size, with a G+C content of 61.5%.

The type strain HLX7-2^T^ (= CGMCC 1.9990^T^=NBRC 116356^T^) was isolated from a cryoconite sample collected from the Hailuogou glacier in Sichuan province, P.R. China. The NCBI accession numbers for the 16S rRNA gene and genome sequences are JX949446 and JBNYXW000000000, respectively.

## Description of *Janthinobacterium salmonicoloratum* sp. nov.

*Janthinobacterium salmonicoloratum* (sal.mo.ni.co.lo.ra’tum. L. masc. n. *salmo*, salmon; L. masc. n. colour, colour; N.L. neut. adj. *salmonicoloratum*, salmon-coloured)

Cells are Gram-stain-negative, aerobic and motile by means of a monopolar flagellum, measuring 1.4–3.7 µm in length and 0.8–1.0 µm in width. Colonies are salmon-coloured, round and slightly convex. Growth occurs at 4–34 °C (optimum, 25–28 °C), pH 4.0–10.0 (optimum, pH 7.0) and 0–2.5% (w/v) NaCl. Positive for oxidase and catalase. Hydrolyse casein, gelatin and aesculin, but not starch and Tween 80. Positive for reduction of nitrate to nitrite, citrate utilization, Voges–Proskauer test, fermentation of glucose, urease, arginine dihydrolase, lysine decarboxylase, ornithine decarboxylase, alkaline phosphatase, esterase (C4), esterase lipase (C8), leucine arylamidase, valine arylamidase, *α*-chymotrypsin, acid phosphatase, naphthol-AS-BI-phosphohydrolase and *α*-glucosidase. Negative for indole production, H_2_S production, *β*-galactosidase, tryptophan deaminase, lipase (C14), cystine arylamidase, trypsin, *α*-galactosidase, *β*-glucuronidase, *β*-glucosidase, *N*-acetyl-*β*-glucosaminidase, *α*-mannosidase and *α*-fucosidase. Utilizes the following carbohydrate as the sole carbon source: glycerol, d-arabinose, l-arabinose, d-ribose, d-xylose, l-xylose, d-galactose, d-glucose, d-fructose, d-mannose, inositol, d-mannitol, d-sorbitol, *N*-acetylglucosamine, arbutin, aesculin, salicin, d-cellobiose, d-maltose, d-lactose, d-sucrose, starch, xylitol, d-lyxose, l-fucose, d-arabitol, gluconate and 2-ketogluconate. Cannot utilize the following carbohydrates: erythritol, d-adonitol, methyl-*β*-d-xylopyranoside, l-sorbose, l-rhamnose, dulcitol, methyl-*α*-d-mannopyranoside, methyl-α-d-glucopyranoside, amygdaline, d-melibiose, d-trehalose, inulin, d-melezitose, d-raffinose, glycogen, gentiobiose, d-turanose, d-tagatose, d-fucose, l-arabitol and 5-ketogluconate. Acids are produced from glycerol, l-arabinose, d-ribose, d-xylose, l-xylose, d-galactose, d-mannose, d-sorbitol, aesculin, d-lyxose and l-fucose. The assembled genome of the type strain is 6.26 Mb in size, with a G+C content of 62.1%.

The type strain LB2P49^T^ (= CGMCC 1.11246^T^=NBRC 116357^T^) was isolated from an ice sample collected from the Laigu glacier on the Tibetan Plateau, P.R. China. The NCBI accession numbers for the 16S rRNA gene and genome sequences are PV277032 and JBNYXV000000000, respectively.

## Description of *Janthinobacterium violaceum* sp. nov.

*Janthinobacterium violaceum* (vi.o.la’ce.um. N.L. neut. adj. *violaceum*, violet-coloured, referring to the colony colour)

Cells are Gram-stain-negative, aerobic and motile by means of a monopolar flagellum, measuring 1.0–2.5 µm in length and 0.8–0.9 µm in width. Colonies are violet-coloured, round and slightly convex. Growth occurs at 4–33 °C (optimum, 25–28 °C), pH 5.0–10.0 (optimum, pH 7.0) and 0–2.5% (w/v) NaCl. Positive for oxidase and catalase. Hydrolyse casein, gelatin and aesculin, but not starch and Tween 80. Positive for reduction of nitrate to nitrite, citrate utilization, Voges–Proskauer test, urease, arginine dihydrolase, lysine decarboxylase, ornithine decarboxylase, alkaline phosphatase, esterase (C4), esterase lipase (C8), leucine arylamidase, valine arylamidase, *α*-chymotrypsin, acid phosphatase, naphthol-AS-BI-phosphohydrolase and *α*-glucosidase. Negative for indole production, H_2_S production, fermentation of glucose, *β*-galactosidase, tryptophan deaminase, lipase (C14), cystine arylamidase, trypsin, *α*-galactosidase, *β-*glucuronidase, *β*-glucosidase, *N*-acetyl-*β*-glucosaminidase, *α*-mannosidase and *α*-fucosidase. Utilizes the following carbohydrate as the sole carbon source: glycerol, d-arabinose, l-arabinose, d-ribose, d-xylose, l-xylose, d-galactose, d-glucose, d-fructose, d-mannose, l-rhamnose, inositol, d-mannitol, d-sorbitol, *N*-acetylglucosamine, arbutin, aesculin, salicin, d-cellobiose, d-maltose, d-lactose, d-sucrose, inulin, d-raffinose, xylitol, d-lyxose, l-fucose, d-arabitol and 2-ketogluconate. Cannot utilize the following carbohydrates: erythritol, d-adonitol, methyl-*β*-d-xylopyranoside, l-sorbose, dulcitol, methyl-*α*-d-mannopyranoside, methyl-*α*-d-glucopyranoside, amygdaline, d-melibiose, d-trehalose, d-melezitose, starch, glycogen, gentiobiose, d-turanose, d-tagatose, d-fucose, l-arabitol, gluconate and 5-ketogluconate. Acids are produced from glycerol, d-arabinose, l-arabinose, d-mannose and aesculin. The assembled genome of the type strain is 6.17 Mb in size, with a G+C content of 62.9%.

The type strain LB2P70^T^ (= CGMCC 1.11252^T^=NBRC 116358^T^) was isolated from an ice sample collected from the Laigu glacier on the Tibetan Plateau, P.R. China. The NCBI accession numbers for the 16S rRNA gene and genome sequences are PV277033 and JBNYXU000000000, respectively.

## Description of *Janthinobacterium leeuwenhoeki* sp. nov.

*Janthinobacterium leeuwenhoeki* (lee.uwen.hoek’i. N.L. gen. n. *leeuwenhoeki*, of Antonie van Leeuwenhoek)

Cells are Gram-stain-negative, aerobic and motile by means of a monopolar flagellum, measuring 1.4–2.2 µm in length and 0.8–1.0 µm in width. Colonies are light yellow-coloured, round and slightly convex. Growth occurs at 4–32 °C (optimum, 25–28 °C), pH 5.0–10.0 (optimum, pH 7.0) and 0–3.0% (w/v) NaCl. Positive for oxidase and catalase. Hydrolyse gelatin and aesculin, but not casein, starch and Tween 80. Positive for reduction of nitrate to nitrite, citrate utilization, Voges–Proskauer test, fermentation of glucose, urease, arginine dihydrolase, ornithine decarboxylase, alkaline phosphatase, esterase (C4), esterase lipase (C8), leucine arylamidase, valine arylamidase, cystine arylamidase, trypsin, *α*-chymotrypsin, acid phosphatase, naphthol-AS-BI-phosphohydrolase, *α*-glucosidase and *β*-glucosidase. Negative for indole production, H_2_S production, *β*-galactosidase, lysine decarboxylase, tryptophan deaminase, lipase (C14), *α*-galactosidase, *β*-glucuronidase, *N*-acetyl-*β*-glucosaminidase, *α*-mannosidase and *α*-fucosidase. Utilizes the following carbohydrate as the sole carbon source: glycerol, l-arabinose, d-ribose, d-galactose, d-glucose, d-fructose, d-mannose, inositol, d-mannitol, d-sorbitol, *N*-acetylglucosamine, d-maltose, d-sucrose, starch, xylitol, d-lyxose, d-arabitol, gluconate and 2-ketogluconate. Cannot utilize the following carbohydrates: erythritol, d-arabinose, d-xylose, l-xylose, d-adonitol, methyl-*β*-d-xylopyranoside, l-sorbose, l-rhamnose, dulcitol, methyl-*α*-d-mannopyranoside, methyl-*α*-d-glucopyranoside, amygdaline, arbutin, aesculin, salicin, d-cellobiose, d-lactose, d-melibiose, d-trehalose, inulin, d-melezitose, d-raffinose, glycogen, gentiobiose, d-turanose, d-tagatose, d-fucose, l-fucose, l-arabitol and 5-ketogluconate. Acids are produced from glycerol, d-galactose, d-glucose, d-mannose and d-sorbitol. The assembled genome of the type strain is 5.63 Mb in size, with a G+C content of 62.0%.

The type strain LB3P112^T^ (= CGMCC 1.11294^T^=NBRC 116359^T^) was isolated from an ice sample collected from the Laigu glacier on the Tibetan Plateau, P.R. China. The NCBI accession numbers for the 16S rRNA gene and genome sequences are PV277034 and JBNYXT000000000, respectively.

## Description of *Janthinobacterium pasteuri* sp. nov.

*Janthinobacterium pasteuri* (pas.teu’ri. N.L. gen. n. *pasteuri*, honouring the French microbiologist Louis Pasteur)

Cells are Gram-stain-negative, aerobic and motile by means of a monopolar flagellum, measuring 1.6–2.6 µm in length and 0.9–1.0 µm in width. Colonies are white-coloured, round and slightly convex. Growth occurs at 4–35 °C (optimum, 25–28 °C), pH 5.0–10.0 (optimum, pH 7.0) and 0–3.0% (w/v) NaCl. Positive for oxidase and catalase. Hydrolyse gelatin and aesculin, but not casein, starch and Tween 80. Positive for reduction of nitrate to nitrite, citrate utilization, Voges–Proskauer test, fermentation of glucose, urease, arginine dihydrolase, alkaline phosphatase, esterase (C4), leucine arylamidase, valine arylamidase, acid phosphatase, naphthol-AS-BI-phosphohydrolase, *α*-glucosidase and *β*-glucosidase. Negative for indole production, H_2_S production, *β*-galactosidase, lysine decarboxylase, ornithine decarboxylase, tryptophan deaminase, esterase lipase (C8), lipase (C14), cystine arylamidase, trypsin, *α*-chymotrypsin, *α*-galactosidase, *β*-glucuronidase, *N*-acetyl-*β*-glucosaminidase, *α*-mannosidase and *α*-fucosidase. Utilizes the following carbohydrate as the sole carbon source: glycerol, l-arabinose, d-ribose, d-galactose, d-glucose, d-fructose, d-mannose, inositol, *N*-acetylglucosamine, arbutin, aesculin, salicin, d-cellobiose, d-maltose, d-lyxose, gluconate and 2-ketogluconate. Cannot utilize the following carbohydrates: erythritol, d-arabinose, d-xylose, l-xylose, d-adonitol, methyl-*β*-d-xylopyranoside, l-sorbose, l-rhamnose, dulcitol, d-mannitol, d-sorbitol, methyl-*α*-d-mannopyranoside, methyl-*α*-d-glucopyranoside, amygdaline, d-lactose, d-melibiose, d-sucrose, d-trehalose, inulin, d-melezitose, d-raffinose, starch, glycogen, xylitol, gentiobiose, d-turanose, d-tagatose, d-fucose, l-fucose, d-arabitol, l-arabitol and 5-ketogluconate. Acids are produced from glycerol, d-galactose, d-mannose and aesculin. The assembled genome of the type strain is 5.68 Mb in size, with a G+C content of 61.8%.

The type strain LB3P118^T^ (= CGMCC 1.11299^T^=NBRC 116360^T^) was isolated from an ice sample collected from the Laigu glacier on the Tibetan Plateau, P.R. China. The NCBI accession numbers for the 16S rRNA gene and genome sequences are PV277035 and JBNYXS000000000, respectively.

## Description of *Janthinobacterium paucivorans* sp. nov.

*Janthinobacterium paucivorans* (pau.ci.vo’rans. L. masc. adj. *paucus*, little; L. pres. part. *vorans*, devouring, eating; N.L. neut. part. adj. *paucivorans*, eating little, referring to the few compounds that are utilized as sole sources of carbon and energy)

Cells are Gram-stain-negative, aerobic and motile by means of a monopolar flagellum, measuring 1.6–2.3 µm in length and 1.0–1.1 µm in width. Colonies are white-coloured, round and slightly convex. Growth occurs at 4–31 °C (optimum, 25–28 °C), pH 5.0–10.0 (optimum, pH 7.0) and 0–2.0% (w/v) NaCl. Positive for oxidase and catalase. Hydrolyse gelatin but not casein, aesculin, starch and Tween 80. Positive for reduction of nitrate to nitrite, citrate utilization, Voges–Proskauer test, urease, arginine dihydrolase, ornithine decarboxylase, alkaline phosphatase, esterase (C4), esterase lipase (C8), leucine arylamidase, valine arylamidase, acid phosphatase, naphthol-AS-BI-phosphohydrolase and *α*-glucosidase. Negative for indole production, H_2_S production, fermentation of glucose, *β*-galactosidase, lysine decarboxylase, tryptophan deaminase, lipase (C14), cystine arylamidase, trypsin, *α*-chymotrypsin, *α*-galactosidase, *β*-glucuronidase, *β*-glucosidase, *N*-acetyl-*β*-glucosaminidase, *α*-mannosidase and *α*-fucosidase. Utilizes the following carbohydrate as the sole carbon source: glycerol, d-ribose, d-glucose, d-fructose, d-mannose, l-rhamnose, inositol, *N*-acetylglucosamine and d-maltose. Cannot utilize the following carbohydrates: erythritol, d-arabinose, l-arabinose, d-xylose, l-xylose, d-adonitol, methyl-*β*-d-xylopyranoside, d-galactose, l-sorbose, dulcitol, d-mannitol, d-sorbitol, methyl-*α*-d-mannopyranoside, methyl-*α*-d-glucopyranoside, amygdaline, arbutin, aesculin, salicin, d-cellobiose, d-lactose, d-melibiose, d-sucrose, d-trehalose, inulin, d-melezitose, d-raffinose, starch, glycogen, xylitol, gentiobiose, d-turanose, d-lyxose, d-tagatose, d-fucose, l-fucose, d-arabitol, l-arabitol, gluconate, 2-ketogluconate and 5-ketogluconate. Acids are produced from glycerol, d-ribose, d-mannose and inositol. The assembled genome of the type strain is 4.90 Mb in size, with a G+C content of 60.8%.

The type strain LB2P10^T^ (= CGMCC 1.11332^T^=NBRC 116361^T^) was isolated from an ice sample collected from the Laigu glacier on the Tibetan Plateau, P.R. China. The NCBI accession numbers for the 16S rRNA gene and genome sequences are LB2P10 and JBNYXR000000000, respectively.

## Description of *Janthinobacterium longum* sp. nov.

*Janthinobacterium longum* (lon’gum. L. neut. adj. *longum*, long)

Cells are Gram-stain-negative, aerobic and motile by means of a monopolar flagellum, measuring 1.5–3.0 µm in length and 0.8–1.0 µm in width. Colonies are light yellow-coloured, round and slightly convex. Growth occurs at 4–35 °C (optimum, 25–28 °C), pH 4.0–10.0 (optimum, pH 7.0) and 0–2.5% (w/v) NaCl. Positive for oxidase and catalase. Hydrolyse aesculin, but not casein, gelatin, starch and Tween 80. Positive for reduction of nitrate to nitrite, citrate utilization, Voges–Proskauer test, fermentation of glucose, urease, *β*-galactosidase, arginine dihydrolase, lysine decarboxylase, ornithine decarboxylase, alkaline phosphatase, esterase (C4), esterase lipase (C8), leucine arylamidase, valine arylamidase, cystine arylamidase, acid phosphatase, naphthol-AS-BI-phosphohydrolase, *α*-glucosidase and *β*-glucosidase. Negative for indole production, H_2_S production, tryptophan deaminase, lipase (C14), trypsin, *α*-chymotrypsin, *α*-galactosidase, *β*-glucuronidase, *N*-acetyl-*β*-glucosaminidase, *α*-mannosidase and *α*-fucosidase. Utilizes the following carbohydrate as the sole carbon source: glycerol, d-arabinose, l-arabinose, d-ribose, d-xylose, d-galactose, d-glucose, d-fructose, d-mannose, l-rhamnose, inositol, d-mannitol, *N*-acetylglucosamine, arbutin, aesculin, salicin, d-cellobiose, d-maltose, d-lactose, d-sucrose, inulin, d-raffinose, d-lyxose, l-fucose and d-arabitol. Cannot utilize the following carbohydrates: erythritol, l-xylose, d-adonitol, methyl-*β*-d-xylopyranoside, l-sorbose, dulcitol, d-sorbitol, methyl-*α*-d-mannopyranoside, methyl-*α*-d-glucopyranoside, amygdaline, d-melibiose, d-trehalose, d-melezitose, starch, glycogen, xylitol, gentiobiose, d-turanose, d-tagatose, d-fucose, l-arabitol, gluconate, 2-ketogluconate and 5-ketogluconate. Acids are produced from glycerol, d-arabinose, l-arabinose, d-ribose, d-xylose, d-galactose, d-glucose, d-fructose, d-mannose, l-rhamnose, inositol, aesculin, d-lyxose and d-arabitol. The assembled genome of the type strain is 5.40 Mb in size, with a G+C content of 61.5%.

The type strain RB2R34^T^ (= CGMCC 1.11894^T^=NBRC 116362^T^) was isolated from an ice sample collected from the Renlongba glacier on the Tibetan Plateau, P.R. China. The NCBI accession numbers for the 16S rRNA gene and genome sequences are PV277037 and JBNYXQ000000000, respectively.

## Description of *Janthinobacterium glycogeni* sp. nov.

*Janthinobacterium glycogeni* (gly.co.ge’ni. N.L. gen. n. *glycogeni*, of glycogen)

Cells are Gram-stain-negative, aerobic and motile by means of a monopolar flagellum, measuring 1.7–2.6 µm in length and 0.9–1.1 µm in width. Colonies are white-coloured, round and slightly convex. Growth occurs at 4–36 °C (optimum, 25–28 °C), pH 4.0–10.0 (optimum, pH 7.0) and 0–3.0% (w/v) NaCl. Positive for catalase and negative for oxidase. Hydrolyse casein, gelatin and aesculin, but not starch and Tween 80. Positive for reduction of nitrate to nitrite, citrate utilization, Voges–Proskauer test, urease, arginine dihydrolase, lysine decarboxylase, ornithine decarboxylase, alkaline phosphatase, esterase (C4), esterase lipase (C8), leucine arylamidase, valine arylamidase, cystine arylamidase, trypsin, acid phosphatase, naphthol-AS-BI-phosphohydrolase, *α*-glucosidase and *β*-glucosidase. Negative for indole production, H_2_S production, fermentation of glucose, *β*-galactosidase, tryptophan deaminase, lipase (C14), *α*-chymotrypsin, *α*-galactosidase, *β*-glucuronidase, *N*-acetyl-*β*-glucosaminidase, *α*-mannosidase and *α*-fucosidase. Utilizes the following carbohydrate as the sole carbon source: glycerol, d-arabinose, l-arabinose, d-ribose, d-xylose, d-galactose, d-glucose, d-fructose, d-mannose, inositol, d-mannitol, d-sorbitol, methyl-*α*-d-mannopyranoside, *N*-acetylglucosamine, arbutin, aesculin, salicin, d-cellobiose, d-maltose, d-lactose, d-sucrose, starch, glycogen, xylitol, d-lyxose, l-fucose, d-arabitol, gluconate and 2-ketogluconate. Cannot utilize the following carbohydrates: erythritol, l-xylose, d-adonitol, methyl-*β*-d-xylopyranoside, l-sorbose, l-rhamnose, dulcitol, methyl-*α*-d-glucopyranoside, amygdaline, d-melibiose, d-trehalose, inulin, d-melezitose, d-raffinose, gentiobiose, d-turanose, d-tagatose, d-fucose, l-arabitol and 5-ketogluconate. Acids are produced from glycerol, d-arabinose, l-arabinose, d-ribose, d-xylose, d-galactose, d-mannose, d-sorbitol, aesculin, d-lyxose and l-fucose. The assembled genome of the type strain is 6.03 Mb in size, with a G+C content of 63.3%.

The type strain ZB1P44^T^ (= CGMCC 1.23234^T^=NBRC 116363^T^) was isolated from an ice sample collected from the Zepu glacier on the Tibetan Plateau, P.R. China. The NCBI accession numbers for the 16S rRNA gene and genome sequences are PV277038 and JBNYXP000000000, respectively.

## Description of *Janthinobacterium kochi* sp. nov.

*Janthinobacterium kochi* (ko’chi. N.L. gen. n. *kochi*, of Robert Koch)

Cells are Gram-stain-negative, aerobic and motile by means of a monopolar flagellum, measuring 1.8–2.5 µm in length and 1.0–1.1 µm in width. Colonies are white-coloured, round and slightly convex. Growth occurs at 4–37 °C (optimum, 25–28 °C), pH 4.0–10.0 (optimum, pH 7.0) and 0–2.5% (w/v) NaCl. Positive for oxidase and catalase. Hydrolyse casein and gelatin but not aesculin, starch and Tween 80. Positive for reduction of nitrate to nitrite, citrate utilization, Voges–Proskauer test, urease, arginine dihydrolase, lysine decarboxylase, ornithine decarboxylase, alkaline phosphatase, esterase (C4), leucine arylamidase, valine arylamidase, acid phosphatase, naphthol-AS-BI-phosphohydrolase and *α*-glucosidase. Negative for indole production, H_2_S production, fermentation of glucose, *β*-galactosidase, tryptophan deaminase, esterase lipase (C8), lipase (C14), cystine arylamidase, trypsin, *α*-chymotrypsin, *α*-galactosidase, *β*-glucuronidase, *β*-glucosidase, *N*-acetyl-*β*-glucosaminidase, *α*-mannosidase and *α*-fucosidase. Utilizes the following carbohydrate as the sole carbon source: glycerol, d-arabinose, l-arabinose, d-ribose, d-xylose, d-galactose, d-glucose, d-fructose, d-mannose, inositol, d-mannitol, d-sorbitol, *N*-acetylglucosamine, d-cellobiose, d-maltose, d-lactose, d-melibiose, d-sucrose, xylitol, d-lyxose, l-fucose, d-arabitol and 2-ketogluconate. Cannot utilize the following carbohydrates: erythritol, l-xylose, d-adonitol, methyl-*β*-d-xylopyranoside, l-sorbose, l-rhamnose, dulcitol, methyl-*α*-d-mannopyranoside, methyl-*α*-d-glucopyranoside, amygdaline, arbutin, aesculin, salicin, d-trehalose, inulin, d-melezitose, d-raffinose, starch, glycogen, gentiobiose, d-turanose, d-tagatose, d-fucose, l-arabitol, gluconate and 5-ketogluconate. Acids are produced from l-arabinose, d-ribose, d-xylose, d-mannitol, d-sorbitol, d-cellobiose, d-maltose, d-melibiose, xylitol, d-lyxose, l-fucose and d-arabitol. The assembled genome of the type strain is 6.06 Mb in size, with a G+C content of 62.2%.

The type strain RB2P8^T^ (= CGMCC 1.23776^T^=NBRC 116364^T^) was isolated from an ice sample collected from the Renlongba glacier on the Tibetan Plateau, P.R. China. The NCBI accession numbers for the 16S rRNA gene and genome sequences are PV277039 and JBNYXO000000000, respectively.

## Description of *Janthinobacterium goodfellowi* sp. nov.

*Janthinobacterium goodfellowi* (good.fel.low’i. N.L. gen. n. *goodfellowi*, of Michael Goodfellow)

Cells are Gram-stain-negative, aerobic and motile by means of a monopolar flagellum, measuring 1.4–2.6 µm in length and 0.9–1.0 µm in width. Colonies are white-coloured, round and slightly convex. Growth occurs at 4–37 °C (optimum, 25–28 °C), pH 4.0–10.0 (optimum, pH 7.0) and 0–2.5% (w/v) NaCl. Positive for oxidase and catalase. Hydrolyse casein, gelatin and aesculin, but not starch and Tween 80. Positive for reduction of nitrate to nitrite, citrate utilization, Voges–Proskauer test, urease, arginine dihydrolase, lysine decarboxylase, ornithine decarboxylase, alkaline phosphatase, esterase (C4), esterase lipase (C8), leucine arylamidase, valine arylamidase, acid phosphatase, naphthol-AS-BI-phosphohydrolase and *α*-glucosidase. Negative for indole production, H_2_S production, fermentation of glucose, *β*-galactosidase, tryptophan deaminase, lipase (C14), cystine arylamidase, trypsin, *α*-chymotrypsin, *α*-galactosidase, *β*-glucuronidase, *β*-glucosidase, *N*-acetyl-*β*-glucosaminidase, *α*-mannosidase and *α*-fucosidase. Utilizes the following carbohydrate as the sole carbon source: glycerol, d-arabinose, l-arabinose, d-ribose, d-xylose, l-xylose, d-galactose, d-glucose, d-fructose, d-mannose, inositol, d-mannitol, d-sorbitol, *N*-acetylglucosamine, arbutin, aesculin, salicin, d-cellobiose, d-maltose, d-lactose, d-sucrose, starch, xylitol, d-lyxose, l-fucose, d-arabitol, gluconate and 2-ketogluconate. Cannot utilize the following carbohydrates: erythritol, d-adonitol, methyl-*β*-d-xylopyranoside, l-sorbose, l-rhamnose, dulcitol, methyl-*α*-d-mannopyranoside, methyl-*α*-d-glucopyranoside, amygdaline, d-melibiose, d-trehalose, inulin, d-melezitose, d-raffinose, glycogen, gentiobiose, d-turanose, d-tagatose, d-fucose, l-arabitol and 5-ketogluconate. Acids are produced from glycerol, l-arabinose, d-ribose, d-xylose, d-galactose, d-glucose, d-fructose, d-mannose, inositol, d-mannitol, d-sorbitol, aesculin, d-cellobiose, d-sucrose, xylitol, d-lyxose, l-fucose and d-arabitol. The assembled genome of the type strain is 6.24 Mb in size, with a G+C content of 63.1%.

The type strain GB1R12^T^ (= CGMCC 1.24272^T^=NBRC 116365^T^) was isolated from an ice sample collected from the Gawalong glacier on the Tibetan Plateau, P.R. China. The NCBI accession numbers for the 16S rRNA gene and genome sequences are PV277040 and JBNYXN000000000, respectively.

## Description of *Janthinobacterium lyxosi* sp. nov.

*Janthinobacterium lyxosi* (ly.xo’si. N.L. gen. n. *lyxosi*, of lyxose)

Cells are Gram-stain-negative, aerobic and motile by means of a monopolar flagellum, measuring 1.4–2.0 µm in length and 0.9–1.0 µm in width. Colonies are light yellow-coloured, round and slightly convex. Growth occurs at 4–30 °C (optimum, 25–28 °C), pH 4.0–10.0 (optimum, pH 7.0) and 0–2.0% (w/v) NaCl. Positive for oxidase and catalase. Hydrolyse gelatin and aesculin, but not casein, starch and Tween 80. Positive for reduction of nitrate to nitrite, citrate utilization, Voges–Proskauer test, fermentation of glucose, arginine dihydrolase, alkaline phosphatase, esterase (C4), leucine arylamidase, valine arylamidase, *α*-chymotrypsin, acid phosphatase, naphthol-AS-BI-phosphohydrolase and *α*-glucosidase. Negative for indole production, H_2_S production, urease, *β*-galactosidase, lysine decarboxylase, ornithine decarboxylase, tryptophan deaminase, esterase lipase (C8), lipase (C14), cystine arylamidase, trypsin, *α*-galactosidase, *β*-glucuronidase, *β*-glucosidase, *N*-acetyl-*β*-glucosaminidase, *α*-mannosidase and *α*-fucosidase. Utilizes the following carbohydrate as the sole carbon source: glycerol, d-arabinose, l-arabinose, d-ribose, d-xylose, d-galactose, d-glucose, d-fructose, d-mannose, l-rhamnose, inositol, *N*-acetylglucosamine, arbutin, aesculin, salicin, d-cellobiose, d-maltose, d-lyxose, d-fucose, l-fucose, gluconate and 2-ketogluconate. Cannot utilize the following carbohydrates: erythritol, l-xylose, d-adonitol, methyl-*β*-d-xylopyranoside, l-sorbose, dulcitol, d-mannitol, d-sorbitol, methyl-*α*-d-mannopyranoside, methyl-*α*-d-glucopyranoside, amygdaline, d-lactose, d-melibiose, d-sucrose, d-trehalose, inulin, d-melezitose, d-raffinose, starch, glycogen, xylitol, gentiobiose, d-turanose, d-tagatose, d-arabitol, l-arabitol and 5-ketogluconate. Acids are produced from glycerol, d-arabinose, l-arabinose, d-ribose, d-xylose, d-galactose, d-mannose, l-rhamnose and aesculin. The assembled genome of the type strain is 6.28 Mb in size, with a G+C content of 62.1%.

The type strain GB4P2^T^ (= CGMCC 1.24307^T^=NBRC 116366^T^) was isolated from an ice sample collected from the Gawalong glacier on the Tibetan Plateau, P.R. China. The NCBI accession numbers for the 16S rRNA gene and genome sequences are PV277041 and JBNYXM000000000, respectively.

## Description of *Janthinobacterium amylolyticum* sp. nov.

*Janthinobacterium amylolyticum* (a.my.lo.ly’ti.cum. Gr. neut. n. *amylon*, starch; N.L. masc. adj. *lyticus*, able to loosen, able to dissolve; from Gr. masc. adj. *lytikos*, able to loosen, dissolving; N.L. neut. adj. *amylolyticum*, starch-dissolving, referring to the property of being able to hydrolyse starch)

Cells are Gram-stain-negative, aerobic and motile by means of a monopolar flagellum, measuring 1.2–2.4 µm in length and 0.9–1.1 µm in width. Colonies are light violet-coloured, round and slightly convex. Growth occurs at 4–37 °C (optimum, 25–28 °C), pH 5.0–10.0 (optimum, pH 7.0) and 0–2.5% (w/v) NaCl. Positive for catalase and negative for oxidase. Hydrolyse starch, gelatin and aesculin, but not casein and Tween 80. Positive for reduction of nitrate to nitrite, citrate utilization, Voges–Proskauer test, urease, arginine dihydrolase, lysine decarboxylase, ornithine decarboxylase, alkaline phosphatase, esterase (C4), leucine arylamidase, valine arylamidase, *α*-chymotrypsin, acid phosphatase, naphthol-AS-BI-phosphohydrolase and *α*-glucosidase. Negative for indole production, H_2_S production, fermentation of glucose, *β*-galactosidase, tryptophan deaminase, esterase lipase (C8), lipase (C14), cystine arylamidase, trypsin, *α*-galactosidase, *β*-glucuronidase, *β*-glucosidase, *N*-acetyl-*β*-glucosaminidase, *α*-mannosidase and *α*-fucosidase. Utilizes the following carbohydrate as the sole carbon source: glycerol, l-arabinose, d-ribose, d-xylose, d-adonitol, d-galactose, d-glucose, d-fructose, d-mannose, inositol, d-mannitol, d-sorbitol, *N*-acetylglucosamine, arbutin, aesculin, salicin, d-cellobiose, d-maltose, d-lactose, starch, xylitol, d-lyxose, d-arabitol, l-arabitol and 2-ketogluconate. Cannot utilize the following carbohydrates: erythritol, d-arabinose, l-xylose, methyl-*β*-d-xylopyranoside, l-sorbose, l-rhamnose, dulcitol, methyl-*α*-d-mannopyranoside, methyl-*α*-d-glucopyranoside, amygdaline, d-melibiose, d-sucrose, d-trehalose, inulin, d-melezitose, d-raffinose, glycogen, gentiobiose, d-turanose, d-tagatose, d-fucose, l-fucose, gluconate and 5-ketogluconate. Acids are produced from glycerol, l-arabinose, d-ribose, d-xylose, d-adonitol, d-galactose, d-glucose, d-fructose, d-mannose, inositol, d-sorbitol, aesculin and d-cellobiose. The assembled genome of the type strain is 6.28 Mb in size, with a G+C content of 62.1%.

The type strain RT4P48^T^ (= CGMCC 1.24355^T^=NBRC 116367^T^) was isolated from a cryoconite sample collected from the Renlongba glacier on the Tibetan Plateau, P.R. China. The NCBI accession numbers for the 16S rRNA gene and genome sequences are PV277042 and JBNYXL000000000, respectively.

## Supplementary material

10.1099/ijsem.0.007020 Uncited Supplementary Material 1.

## Abbreviations

ANI: average nucleotide identity
COG: Clusters of Orthologous Groups
dDDH: digital DNA–DNA hybridization
MAGs: metagenome-assembled genomes
ML: maximum likelihood
NJ: neighbour-joining
PYG: peptone, yeast extract and glucose
