# Supplementary material for: Description of 15 novel species within genus Janthinobacterium isolated from glaciers
Source: Int J Syst Evol Microbiol. 2026 Jan 14;76(1):007020. doi: 10.1099/ijsem.0.007020 (PMC12804093; doi:10.1099/ijsem.0.007020)
Supplement: Uncited Supplementary Material 1. [file ijsem-76-07020-s001.pdf]

Description of fifteen novel species within genus *Janthinobacterium* isolated from glaciers

Lei-Lei Yang, Yu-Hua Xin, Qing Liu

International Journal of Systematic and Evolutionary Microbiology

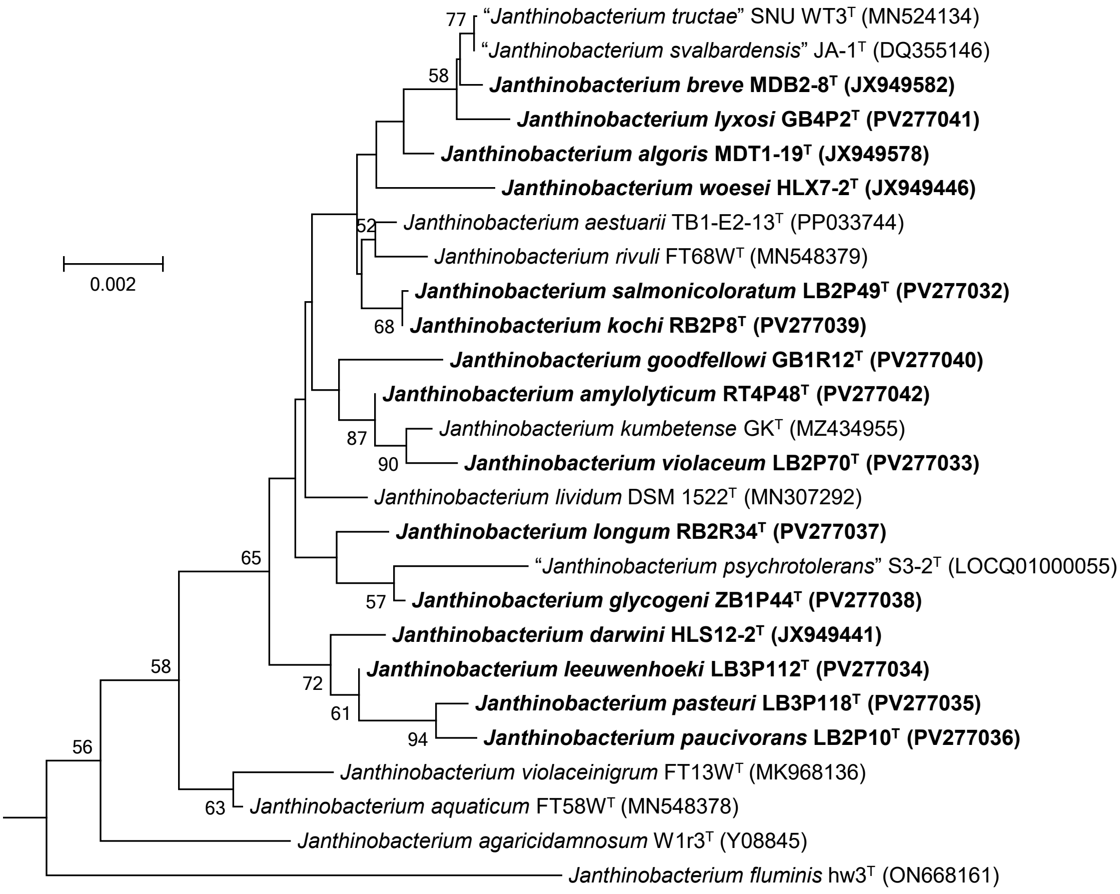

Fig. S1. Phylogenetic tree of the 15 strains and related taxa based on the 16S rRNA gene sequence comparisons using the NJ method. GenBank accession numbers of the 16S rRNA gene sequences are given in parentheses. Bootstrap values (>50 %) based on 1,000 replicates are shown at the branch nodes. Bar, 0.002 substitutions per nucleotide positions.

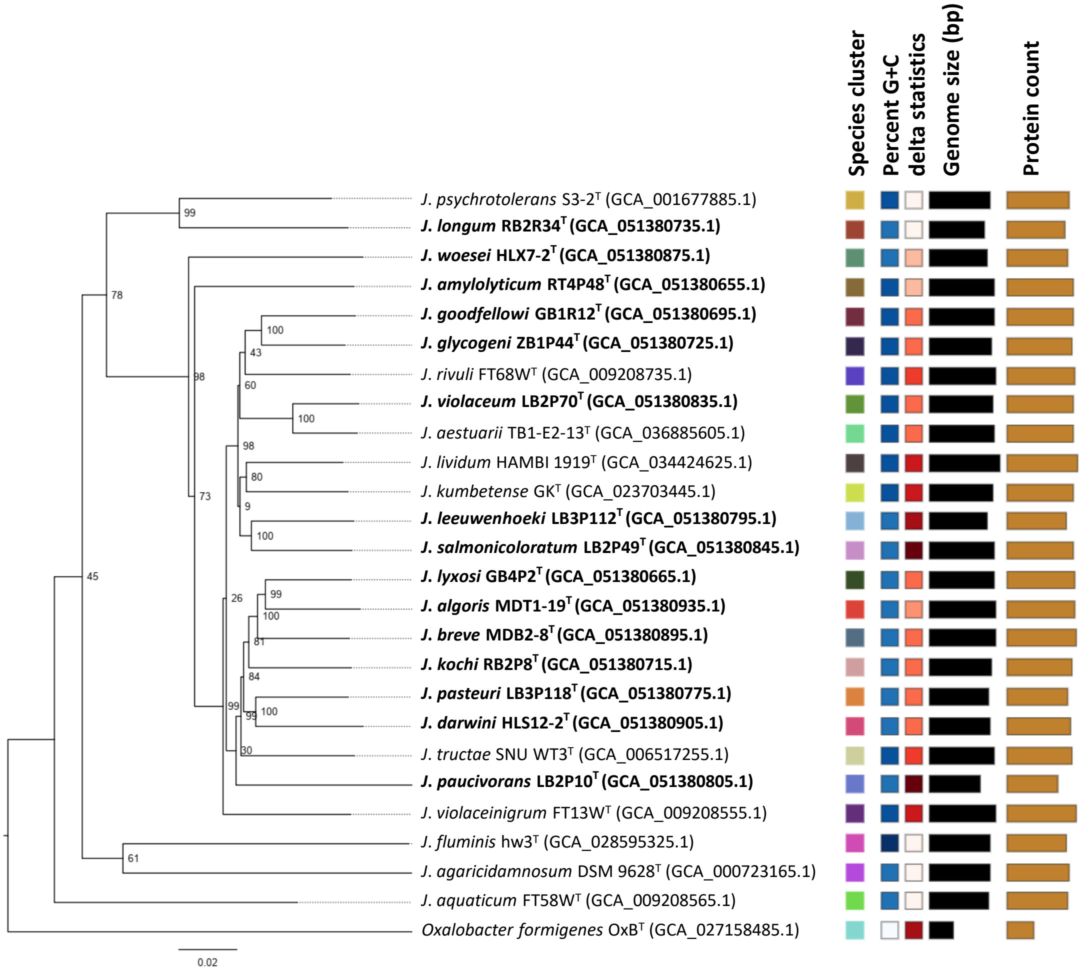

Fig. S2. Genome BLAST distance phylogeny (GBDP) of selected genomes, inferred using the TYGS webserver. Node values are based on 100 pseudo-bootstrap replicates. Branch lengths are scaled in terms of GBDP distance formula *d4*.

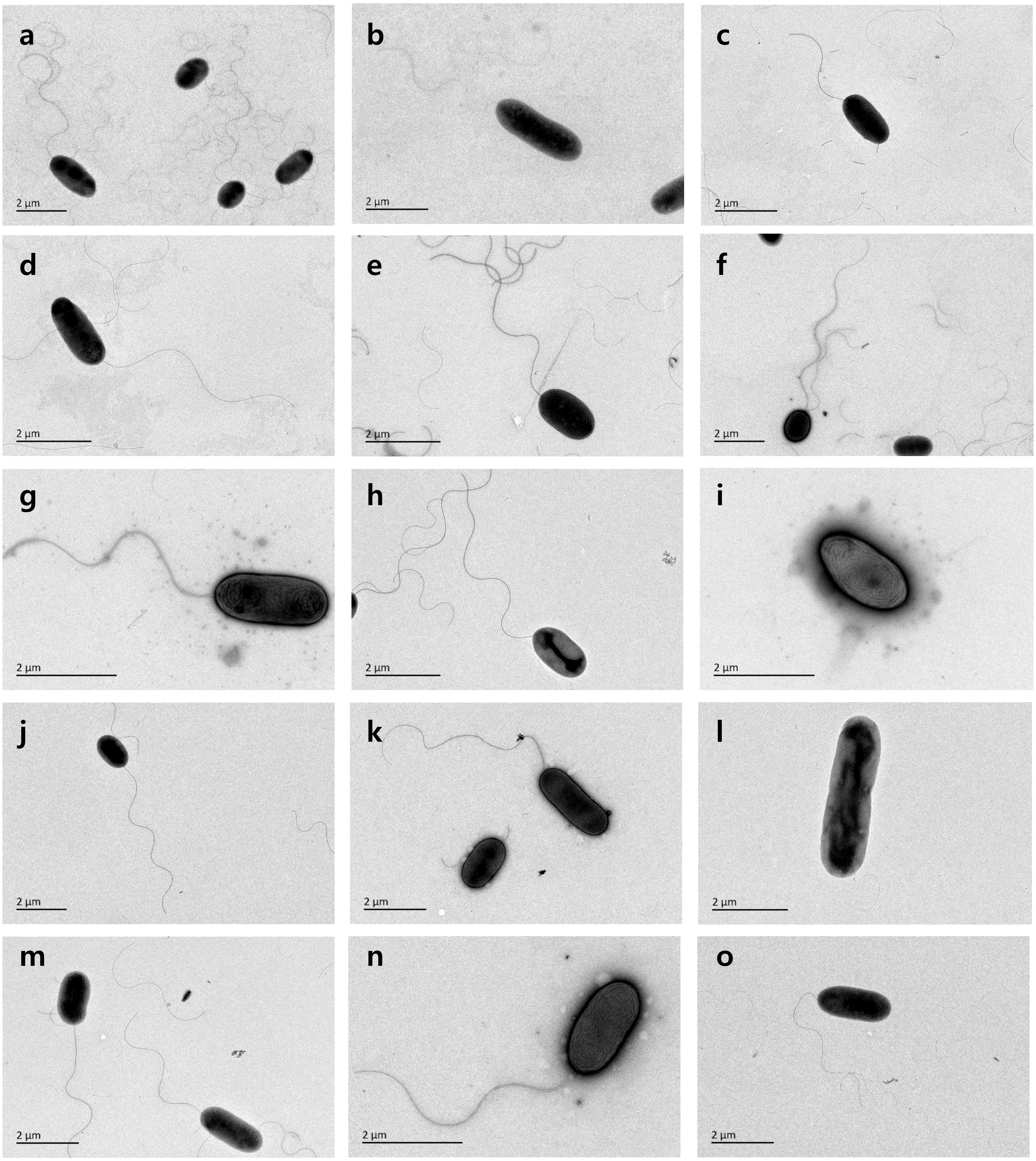

Fig. S3. Transmission electron micrograph of negatively stained cells of strains MDT1-19^T (a), MDB2-8^T (b), HLS12-2^T (c), HLX7-2^T (d), LB2P49^T (e), LB2P70^T (f), LB3P112^T (g), LB3P118^T (h), LB2P10^T (i), RB2R34^T (j), ZB1P44^T (k), RB2P8^T (l), GB1R12^T (m), GB4P2^T (n), and RT4P48^T (o) grown at 25°C on PYG agar. Scale bar, 2 μm.

Table S1. Origin and sampling information of the 15 isolates

Strain	Glacier	Sample type	Elevation (m a.s.l.)	Longitude (°E)	Latitude (°N)	Sampling date
MDT1-19^T	Midui glacier	Cryoconite	3901	96.505000	29.451944	Oct 2010
MDB2-8^T	Midui glacier	Cryoconite	3901	96.505000	29.451944	Oct 2010
HLS12-2^T	Hailuogou glacier	Cryoconite	3457	101.968611	29.555833	Jan 2011
HLX7-2^T	Hailuogou glacier	Cryoconite	3457	101.968611	29.555833	Jan 2011
LB2P49^T	Laigu glacier	Ice	3931.6	96.8186951	29.308783	Oct 2016
LB2P70^T	Laigu glacier	Ice	3931.6	96.8186951	29.308783	Oct 2016
LB3P112^T	Laigu glacier	Ice	3931.6	96.8186951	29.308783	Oct 2016
LB3P118^T	Laigu glacier	Ice	3931.6	96.8186951	29.308783	Oct 2016
LB2P10^T	Laigu glacier	Ice	3931.6	96.8186951	29.308783	Oct 2016
RB2R34^T	Renlongba glacier	Ice	4651.7	96.9359436	29.261593	Oct 2016
ZB1P44^T	Zepu glacier	Ice	3454.6	95.2508392	30.276556	Oct 2016
RB2P8^T	Renlongba glacier	Ice	4651.7	96.9359436	29.261593	Oct 2016
GB1R12^T	Gawalong glacier	Ice	3842.3	95.710350	29.765926	Oct 2016
GB4P2^T	Gawalong glacier	Ice	3842.3	95.710350	29.765926	Oct 2016
RT4P48^T	Renlongba glacier	Cryoconite	4651.7	96.935944	29.261593	Oct 2016

Table S2. ANI values and 16S rRNA gene sequence similarity (%) between the 15 strains isolated in this study

ANI/16S rRNA gene sequence similarity	1	2	3	4	5	6	7	8	9	10	11	12	13	14	15
1. MDT1-19^{T}		99.78	99.56	99.71	99.78	99.63	99.56	99.71	99.56	99.56	98.90	99.78	99.48	99.70	99.63
2. MDB2-8^{T}	94.03		99.78	99.48	99.63	99.56	99.63	99.71	99.56	99.56	99.05	99.63	99.41	99.85	99.56
3. HLS12-2^{T}	92.41	93.04		99.41	99.70	99.63	99.93	99.78	99.85	99.63	99.71	99.70	99.41	99.62	99.63
4. HLX7-2^{T}	89.41	89.88	89.53		99.63	99.48	99.41	99.41	99.41	99.41	99.26	99.63	99.33	99.40	99.48
5. LB2P49^{T}	92.80	93.10	92.63	89.74		99.71	99.70	99.56	99.56	99.63	99.05	100.0	99.56	99.62	99.63
6. LB2P70^{T}	91.76	92.03	91.78	89.68	92.87		99.70	99.71	99.71	99.78	99.19	99.71	99.70	99.47	99.93
7. LB3P112^{T}	92.42	92.61	92.62	89.83	93.71	92.71		99.85	99.85	99.70	99.63	99.70	99.48	99.63	99.70
8. LB3P118^{T}	93.14	93.95	93.81	89.63	92.75	91.97	92.97		99.85	99.56	98.90	99.56	99.48	99.63	99.63
9. LB2P10^{T}	90.37	90.79	90.64	88.21	90.10	89.77	90.34	90.83		99.56	98.90	99.56	99.48	99.47	99.71
10. RB2R34^{T}	85.03	85.29	85.10	85.40	85.35	85.52	85.41	85.20	84.47		99.12	99.63	99.48	99.55	99.71
11. ZB1P44^{T}	91.95	92.29	92.09	89.81	92.88	93.25	92.67	92.15	89.98	85.59		99.05	99.48	99.40	99.49
12. RB2P8^{T}	93.53	93.74	92.77	89.70	92.97	92.35	93.13	93.52	90.65	85.26	92.46		99.56	99.62	99.71
13. GB1R12^{T}	91.95	92.34	92.17	89.97	92.92	93.20	92.70	92.35	90.04	85.71	94.76	92.39		99.32	99.70
14. GB4P2^{T}	94.26	94.23	92.81	89.86	93.49	92.20	92.99	93.54	90.74	85.27	92.40	93.78	92.41		99.47
15. RT4P48^{T}	89.97	90.30	90.05	89.83	90.57	91.04	90.68	90.24	88.46	85.66	90.91	90.33	90.95	90.30	

Table S3. The basic information and the BLAST comparison result using 16S rRNA gene sequences of the 15 strains

Strain	CGMCC NO.	NBRC NO.	16S rDNA accession no.	Closest relatives	16S rRNA gene sequence similarity(%)
MDT1-19^{T}	1.9797	116353	JX949578	"*Janthinobacterium tructae*" SNU WT3^{T}	99.86
MDB2-8^{T}	1.9853	116354	JX949582	"*Janthinobacterium svalbardensis*" JA-1^{T}	99.93
HLS12-2^{T}	1.9980	116355	JX949441	*Janthinobacterium lividum* DSM 1522^{T}	99.71
HLX7-2^{T}	1.9990	116356	JX949446	*Janthinobacterium rivuli* FT68W^{T}	99.64
LB2P49^{T}	1.11246	116357	PV277032	*Janthinobacterium rivuli* FT68W^{T}	99.71
LB2P70^{T}	1.11252	116358	PV277033	*Janthinobacterium lividum* DSM 1522^{T}	99.78
LB3P112^{T}	1.11294	116359	PV277034	*Janthinobacterium lividum* DSM 1522^{T}	99.71
LB3P118^{T}	1.11299	116360	PV277035	"*Janthinobacterium svalbardensis*" JA-1^{T}	99.78
LB2P10^{T}	1.11332	116361	PV277036	*Janthinobacterium lividum* DSM 1522^{T}	99.20
RB2R34^{T}	1.11894	116362	PV277037	"*Janthinobacterium svalbardensis*" JA-1^{T}	99.57
ZB1P44^{T}	1.23234	116363	PV277038	"*Janthinobacterium psychrotolerans*" S3-2^{T}	99.72
RB2P8^{T}	1.23776	116364	PV277039	*Janthinobacterium rivuli* FT68W^{T}	99.20
GB1R12^{T}	1.24272	116365	PV277040	*Janthinobacterium kumbetense* GK^{T}	99.49
GB4P2^{T}	1.24307	116366	PV277041	"*Janthinobacterium tructae*" SNU WT3^{T}	99.85
RT4P48^{T}	1.24355	116367	PV277042	*Janthinobacterium kumbetense* GK^{T}	99.93

Table S4. Basic genome information for the 15 strains isolated in this study

Strain	Proposal name	Genome size	GC (%)	Completeness (%)	Contamination (%)	Contigs	N50 (Mb)	gaps
MDT1-19^T	*Janthinobacterium algoris*	6.40	61.6	100	0.03	48	0.28	5
MDB2-8^T	*Janthinobacterium breve*	6.44	61.9	100	0.11	52	0.45	3
HLS12-2^T	*Janthinobacterium darwini*	5.85	61.9	100	0.34	15	3.35	2
HLX7-2^T	*Janthinobacterium woesei*	5.63	61.5	100	0	56	0.27	0
LB2P49^T	*Janthinobacterium salmonicoloratum*	6.26	62.1	100	0.03	20	0.79	0
LB2P70^T	*Janthinobacterium violaceum*	6.17	62.9	100	0.05	25	0.46	3
LB3P112^T	*Janthinobacterium leeuwenhoeki*	5.63	62.0	100	0.03	78	0.12	2
LB3P118^T	*Janthinobacterium pasteuri*	5.68	61.8	100	0.28	54	0.17	2
LB2P10^T	*Janthinobacterium paucivorans*	4.90	60.8	99.99	0.11	42	0.25	2
RB2R34^T	*Janthinobacterium longum*	5.40	61.5	100	0.01	25	0.44	1
ZB1P44^T	*Janthinobacterium glycogeni*	6.03	63.3	100	0.05	31	0.47	1
RB2P8^T	*Janthinobacterium kochi*	6.06	62.2	100	0.04	38	0.45	1
GB1R12^T	*Janthinobacterium goodfellowi*	6.24	63.1	100	0.18	21	0.80	4
GB4P2^T	*Janthinobacterium lyxosi*	6.28	62.1	100	1.38	88	0.22	1
RT4P48^T	*Janthinobacterium amylolyticum*	6.24	63.1	100	0.39	30	0.44	1

Table S5. Basic information on genome annotation for the 15 strains

	CRISPR arrays	Coding density	CDSs	ncRNA regions	ncRNAs	rRNAs	tRNAs	tmRNA	Hypotheticals	Pseudogenes
MDT1-19^T	0	89.5	5600	12	13	7	79	1	468	26
MDB2-8^T	0	89.8	5664	14	15	7	81	1	357	19
HLS12-2^T	0	89.9	5233	13	12	7	80	1	271	15
HLX7-2^T	0	89.4	4971	13	13	5	79	1	324	18
LB2P49^T	0	90.5	5474	14	12	4	76	1	281	11
LB2P70^T	0	90.6	5446	12	13	6	81	1	238	14
LB3P112^T	0	89.8	4923	11	13	7	79	1	276	19
LB3P118^T	0	89.5	5018	12	11	6	80	1	345	18
LB2P10^T	0	88.9	4225	11	10	7	78	1	263	9
RB2R34^T	0	90.8	4780	11	13	5	75	1	336	8
ZB1P44^T	0	90.4	5326	14	14	8	79	1	202	16
RB2P8^T	0	90.2	5323	15	12	9	81	1	318	15
GB1R12^T	0	90.5	5506	12	12	9	80	1	261	11
GB4P2^T	0	89.6	5628	14	11	7	85	1	458	38
RT4P48^T	0	89.4	5466	13	11	6	80	1	296	4

Table S6. Differential characteristic phenotype of strains LB2P70^T and *Janthinobacterium aestuarii* TB1-E2-13^T. *, data was cited from Pavloudi *et al.* [4]

	**LB2P70^T**	**TB1-E2-13^T***
pH range	5–10	5.5–8.0
NaCl concentration range for growth (%)	0–2.5	0–1
Growth temperature (°C)	26-33	10–40
Hydrolysis of casein	+	-
Voges-Proskauer test	+	-
Urease	+	-
Utilization of D-fucose	+	-
